# Generalized Pesin-Like Identity and Scaling Relations at the Chaos Threshold of the Rössler System

**DOI:** 10.3390/e20040216

**Published:** 2018-03-23

**Authors:** Kivanc Cetin, Ozgur Afsar, Ugur Tirnakli

**Affiliations:** Department of Physics, Faculty of Science, Ege University, 35100 Izmir, Turkey

**Keywords:** nonlinear dynamics, connections between chaos and statistical physics, dissipative systems

## Abstract

In this paper, using the Poincaré section of the flow we numerically verify a generalization of a Pesin-like identity at the chaos threshold of the Rössler system, which is one of the most popular three-dimensional continuous systems. As Poincaré section points of the flow show similar behavior to that of the logistic map, for the Rössler system we also investigate the relationships with respect to important properties of nonlinear dynamics, such as correlation length, fractal dimension, and the Lyapunov exponent in the vicinity of the chaos threshold.

## 1. Introduction

In many disciplines, continuous systems are used for modeling systems to ease operation and obtain a better viewpoint with respect to dynamics. In addition to applications in physics [1,2], biology [3,4], and chemistry [5] etc., continuous systems are widely used in nonlinear dynamics studies as test systems [6,7,8,9]. As three-dimensional dissipative continuous systems can exhibit chaotic behavior according to their parameter values, these systems have become more popular recently [10,11,12,13]. The period-doubling bifurcations that are seen in model systems which exhibit a transition from periodic to chaotic behavior [14,15] can also be seen in nature, and in experiments involving for example semiconductors [16] and RLC circuits [17]. A good example of these systems is the Rössler system, which can exhibit complex dynamics and chaos. Although it was constructed as a simple system to obtain chaotic behavior [18], the Rössler system has become a test system for new numerical techniques [7,8]. The Rössler system is modeled with three ordinary differential equations given by

(1) $$\begin{array}{c} \dot{x}=-y-z\\ \dot{y}=x+ay\\ \dot{z}=b+z\left(x-c\right) \end{array}$$

where *a*, *b*, and *c* are the control parameters that are assumed to be positive and dimensionless [19].

Traditionally, two of three control parameters are kept constant and the effects of changing the last control parameter on the system are observed. In our investigations we keep a=b=0.2 as constant parameters, and we use *c* as a control parameter. While the *a* and *b* parameters are kept constant and *c* varies from zero to larger values, the Rössler system changes its periodic behavior to chaotic after *c* reaches the critical point $c_{c}$, the chaos threshold. Above this chaos threshold there also exist periodic windows in the chaotic region.

**Figure 1:**
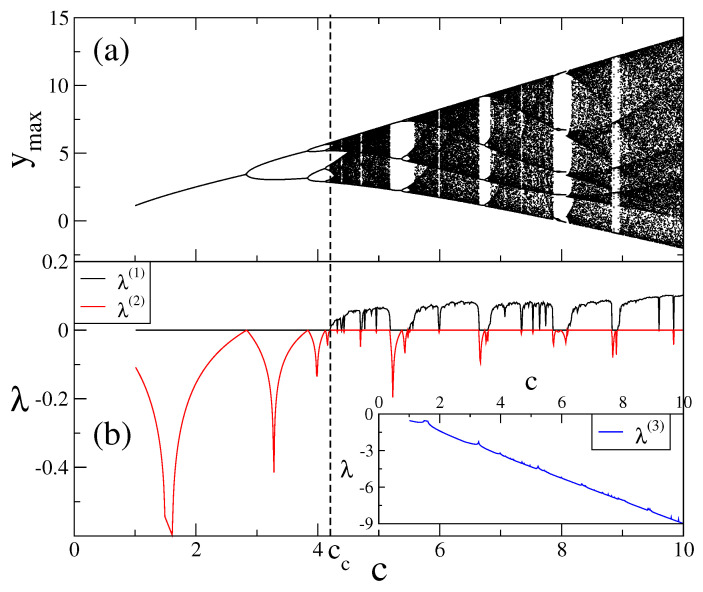
(**a**) Bifurcation diagram obtained from the Poincaré section intersection points of the *y* variable of the Rössler system; (**b**) the Lyapunov exponent diagram of the Rössler system starting from initial points x=y=z=1, with constant parameters a=b=0.2.

As the three-dimensional state space of the Rössler system is complicated for our analysis, here the use of the Poincaré section reduces the dimensions of the state space that we investigate, and through an appropriate choice of the Poincaré section we can obtain a full picture of system dynamics [20]. In our investigations, the Poincaré section is constructed by collecting maximum points of a signal of a variable of the Rössler system. With that choice of Poincaré section, we reduce the dimensions of the state space by two and we are able to investigate the Rössler system as one-dimensional. In Figure 1a we see that the system becomes chaotic after successive period doublings which terminate at the chaos threshold where the system has an infinite number of periods. This bifurcation diagram is in the same universality class as the one-dimensional dissipative logistic map [20].

In Figure 1b we plot the Lyapunov exponent diagram as a descriptor of bifurcation diagram. The three Lyapunov exponents $\lambda^{\left(1\right)}$, $\lambda^{\left(2\right)}$, and $\lambda^{\left(3\right)}$, are the characteristic exponents of the state space, and they are the eigenvalues of the Jacobian matrix

(2) $$\begin{array}{c} J=\left(\begin{array}{ccc} 0 & -1 & -1\\ 1 & a & 0\\ z & 0 & x-c \end{array}\right) \end{array}$$

constructed for the Rössler system. As the Lyapunov exponent is a measure of the average convergence or divergence of neighbor trajectories in the state space [20], we see that the combination of $\lambda^{\left(1\right)}$ and $\lambda^{\left(2\right)}$ explains behaviors that we observe at the bifurcation diagram. $\lambda^{\left(3\right)}$ becomes more negative as the *c* parameter increases, so we plot this exponent in an inner plot apart from other two. We detect the chaos threshold of the Rössler system as $c_{c}=4.204232...$.

For the strongly chaotic regime it can be seen from Figure 1b that the Lyapunov exponent is positive. For this exponent value, the initially nearby trajectories diverge in an exponential form as the system evolves. As trajectories spread into the state space, the relation between the Lyapunov exponent and the entropy increase rate, namely Kolmogorov–Sinai (KS) entropy, is given by the Pesin identity [21]. This states that for the strongly chaotic case, the Lyapunov exponent and KS entropy should be equal, whereas for the periodic case, KS entropy should be zero. In this paper, at the chaos threshold of the Rössler system where the Lyapunov exponent is zero and the behavior is weakly chaotic, we numerically investigate a generalization of a Pesin-like identity similar to that of the logistic map, which has been shown recently in [22,23,24,25]. We also investigate the scaling relations among the correlation length, the Lyapunov exponent, and the fractal dimension in the vicinity of the chaos threshold for the Rössler system.

## 2. Sensitivity to the Initial Conditions, Entropy Increase Rate, and the Pesin Identity at the Chaos Threshold

One of the signatures of the chaotic behavior is exponential sensitivity to the initial conditions. This sensitivity property is characterized by the sensitivity function given by

(3) $$\xi \left(t\right)\equiv \lim_{\Delta x(0)\to 0}\frac{\Delta x(t)}{\Delta x(0)}$$

for a one-dimensional case, where Δx(0) is the initial distance between two nearby points and Δx(t) is the distance at time *t* [20]. If the system has a positive Lyapunov exponent, which corresponds to the chaotic behavior, the sensitivity function diverges as $\xi \left(t\right)=\exp\left(\lambda_{1}t\right)$, where $\lambda_{1}$ is the standard Lyapunov exponent (subindex will be explained later). For $\lambda_{1}>0$ the system is said to be strongly sensitive and for $\lambda_{1}<0$ strongly insensitive to the initial conditions. At the chaos threshold the standard Lyapunov exponent vanishes ($\lambda_{1}=0$) and for this marginal case it has been shown for several dissipative maps that the sensitivity function exhibits power-law behavior of the form $\xi \left(t\right)=\exp_{q}\left(\lambda_{q}t\right)$, where $\lambda_{q}$ is the generalized Lyapunov exponent and

(4) $$\exp_{q}\left(x\right)={\left[1+\left(1-q\right)x\right]}^{\frac{1}{1-q}}$$

which is known to be *q*-exponential [22,23,24,25]. This expression recovers the standard exponential case as q→1, namely, $\exp_{1}\left(x\right)=\exp\left(x\right)$. Thus, the sensitivity function can be given by

(5) $$\xi \left(t\right)={\left[1+\left(1-q\right)\lambda_{q}t\right]}^{\frac{1}{1-q}}$$

in the vicinity of the chaos threshold. For $\lambda_{q}>0$ and q<1 ($\lambda_{q}<0$ and q>1), the system is said to be weakly sensitive (weakly insensitive) to the initial conditions [22]. At the chaos threshold, a *q* value that yields power-law behavior for ξ is always obtained for q<1 [25].

For chaotic systems, another way to describe the behavior is through KS entropy $K_{1}$ which is the increase, per unit time, of the Boltzmann–Gibbs (BG) entropy ($S_{BG}\equiv -\sum_{i=1}^{W}p_{i}\ln p_{i}$, where *W* is the total number of possible configurations and $p_{i}$ is the probability of finding a point in the *i*th state) and it is related to the standard Lyapunov exponent via the Pesin identity [21], which states that if $\lambda_{1}>0$, $K_{1}=\lambda_{1}$ and otherwise $K_{1}=0$. Although KS entropy can be defined in terms of a single trajectory in a phase space using a symbolic representation of cells of a partitioned phase space [20], in almost all cases this definition can be replaced by a version based on an ensemble of initial conditions [24,25,26]. In this paper we use this version.

As BG entropy requires strong chaos for its applicability, the *q*-entropy $S_{q}$ that is the basis of the non-extensive statistical mechanics [22,27,28] can recover both $\lambda_{1}>0$ chaotic and $\lambda_{1}=0$ marginal cases. This entropy is given as

(6) $$S_{q}\equiv \frac{1-\sum_{i=1}^{W}p_{i}^{q}}{q-1}=\sum_{i=1}^{W}p_{i}\ln_{q}\left(\frac{1}{p_{i}}\right)$$

where $\ln_{q}\left(x\right)$ is the *q*-logarithm function that is the inverse of the *q*-exponential given in Equation (4). As q→1 this function becomes the standard logarithm function $\ln_{1}\left(x\right)=\ln\left(x\right)$ and the entropy achieves the BG case. From this perspective, KS entropy can also be generalized as [22]

(7) $$K_{q}\equiv \lim_{t\to \infty }\lim_{W\to \infty }\lim_{N\to \infty }\frac{S_{q}\left(t\right)}{t}$$

where *t* is the time steps, *N* is the number of points, and *W* is the number of regions in the partitioned phase space. With these generalizations the Pesin identity can also be generalized by including the standard case as a special case as $K_{q}=\lambda_{q}$ for $\lambda_{q}>0$ and $K_{q}=0$ otherwise [22].

**Figure 2:**
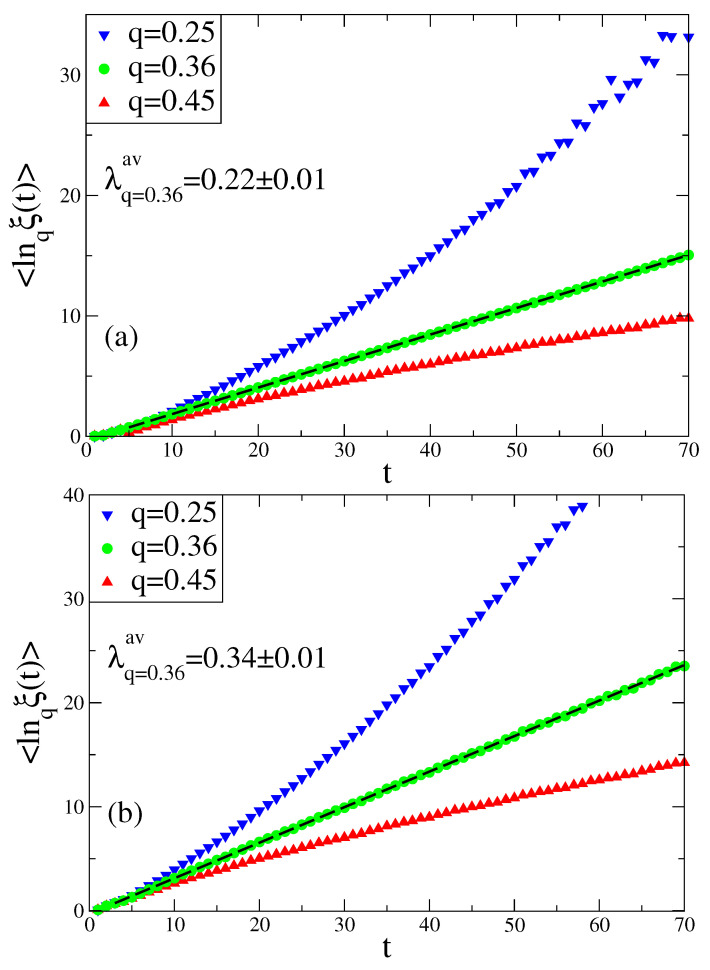

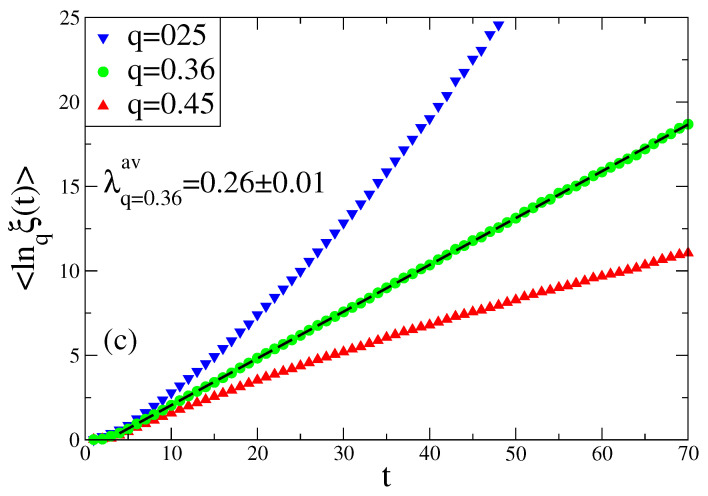
Time evolution of the averaged sensitivity function at the chaos threshold of the Rössler system for the (**a**) *x* , (**b**) *y* , and (**c**) *z* variables.

In our investigations on the sensitivity to the initial conditions at the chaos threshold of the Rössler system we use the sensitivity function given by Equation (3) and use $\Delta x\left(0\right)=10^{-12}$ as an initial separation of two nearby points in the phase space. Starting from these points we let the system evolve and then we collect Poincaré points of the flow. This procedure is repeated for $5\times 10^{7}$ randomly chosen different initial conditions to make an ensemble average. For Poincaré points of each variable of the Rössler system, an average is taken all over those values, namely, $\langle \ln_{q}\xi \left(t\right)\rangle$. In Figure 2, for a specific *q* value ($q_{sen}^{av}=0.36$), we observe a linear time dependence of $\langle \ln_{q}\xi \left(t\right)\rangle$ and the slope of this curve gives the generalized Lyapunov exponent $\lambda_{sen}^{av}$ from Equation (5).

At the chaos threshold, we analyze generalized KS entropy using Equation (6). As entropy calculation requires dividing the phase space into *W* small cells, putting *N* randomly chosen initial conditions into a cell, and letting the system evolve, in order to obtain a reasonable behavior of Poincaré points of a variable of the Rössler system, our method is described in three steps. Firstly, for a variable for which we investigate its Poincaré points, we divide the one-dimensional phase space obtained by the Poincaré section into $W=2\times 10^{4}$ equal intervals and keep other two variables at a constant value that is used to obtain the bifurcation diagram given in Figure 1a. Since the Rössler system is continuous, it is not possible to divide all of the phase space. In our investigations, using the dissipative nature of the system, we divide the region at a point between zero and slightly above the maximum of the chaos threshold phase space attractor, which we see in the bifurcation diagram. With this region selection we can also observe transient behavior as the system reaches its attractor. Secondly, we put N=10W randomly chosen initial conditions into one of the equally-sized intervals. Then, we let the system evolve for each of these *N* points and collect Poincaré points. As the flow intersects with the Poincaré section, these points occupy *W* intervals, and relative frequency $p_{i}\left(t\right)\equiv N_{i}\left(t\right)/N$ [20] can be calculated where $N_{i}\left(t\right)$ is the occupancy ($\sum_{i=1}^{W}N_{i}\left(t\right)=N$). In this formalism *t* corresponds to the intersection step in the Poincaré section. Thirdly, with the relative frequency we can calculate $S_{q}$ and the entropy production per unit time $K_{q}$. We repeat this procedure many times starting from randomly chosen W/2 different intervals to make an ensemble average.

**Figure 3:**
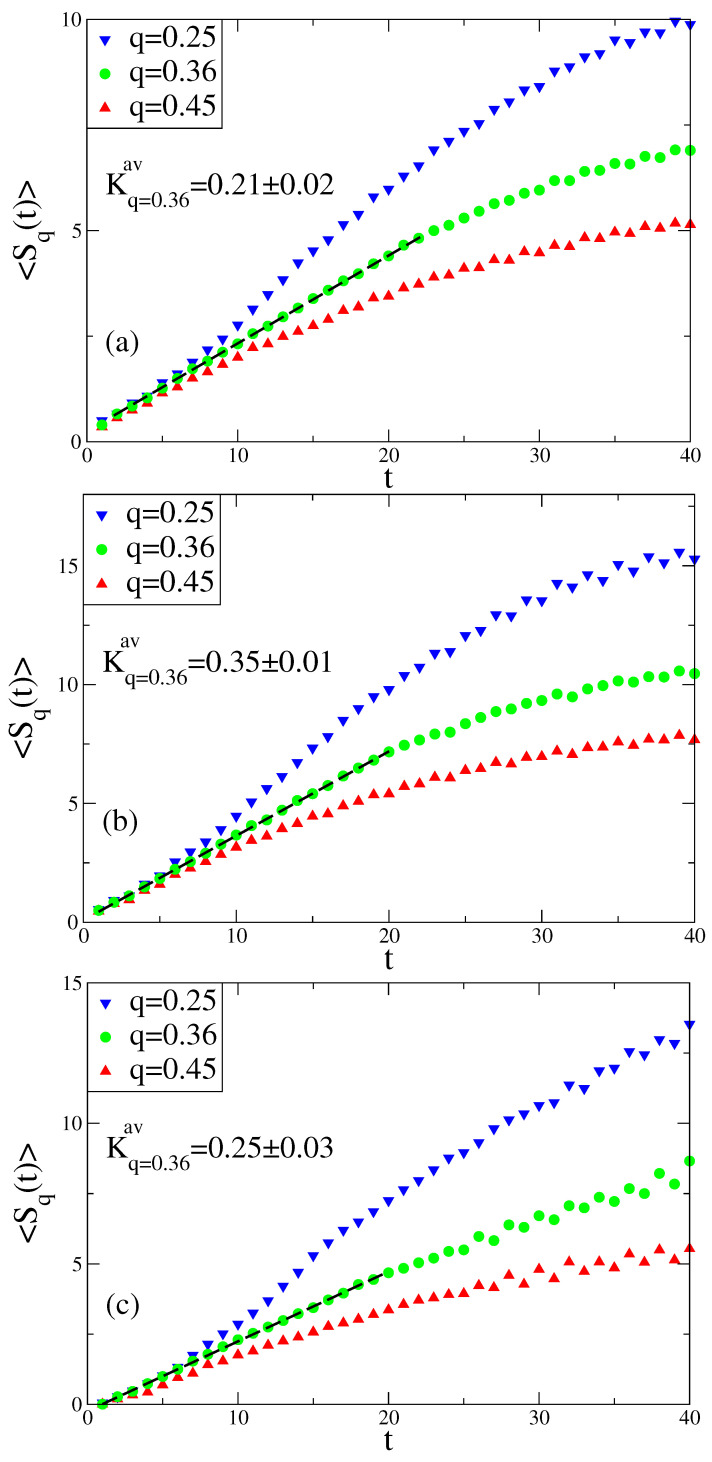
Entropy increase rates at the chaos threshold of the Rössler system for the (**a**) *x* , (**b**) *y* , and (**c**) *z* variables.

In recent works [25,29] it has been shown that $K_{q}$ is finite only for $q=q_{sen}^{av}$, while $K_{q}$ diverges and is equal to zero for $q<q_{sen}^{av}$ and $q>q_{sen}^{av}$, respectively. In Figure 3 it can be seen that we obtain a linear increase of the $S_{q}$ and $K_{sen}^{av}$ values, showing the slope of this linear curve that coincides with $\lambda_{sen}^{av}$ (with numerical error). One can argue that this *q*-generalization of the Pesin-like identity holds for the Rössler system at the chaos threshold. In Figure 3, linear regions are detected using the maximum R-squared value, which is ≃0.999 for three plots. Above this region we see the initiation of the saturation due to the finiteness of *W*.

## 3. Scaling Relations of the Rössler System at the Edge of Chaos

As the Poincaré section points of the Rössler system show the power-law behavior for the sensitivity to the initial conditions at the chaos threshold, we can examine other power-law behaviors obtained for scaling relations in the vicinity of the chaos threshold as done recently for the logistic map [30,31]. To investigate scaling relations for the Rössler system, we use the generalized Huberman–Rudnick scaling law to get closer to the chaos threshold from the chaotic region [32]. This generalized scaling law is defined as

(8) $$\left|c-c_{c}^{\left(s\right)}\right|=\delta^{-n-\frac{\ln s}{\ln2}}$$

where δ=4.669201... is one of the Feigenbaum constants, $c_{c}$ is the chaos threshold parameter value, *s* is a parameter describing each particular period window, and *n* is an index that represents the $s2^{n}$ chaotic bands (n=1,2,...,∞) which approach the Feigenbaum attractor as n→∞ by the band-splitting procedure [32]. Here, we only focus on the period-2 (s=1) window. As the chaos threshold is approached with increasing *n* values, we take the control parameter values *c* corresponding to these *n* values. Increasing *n* values with the same precision makes us approach the chaos threshold by staying on one of the infinitely many possible straight lines, all having positive Lyapunov exponents, given by the Huberman-Rudnick scaling law. As the Lyapunov exponents for integer *n* values tend to -∞, we choose non-integer *n* values and increase them with the same precision. These parameter values are listed in Table 1. By using (n,c) tuples, we numerically investigate scaling relations as the chaos threshold is approached.

### 3.1. Huberman–Rudnick Scaling

**Figure 4:**
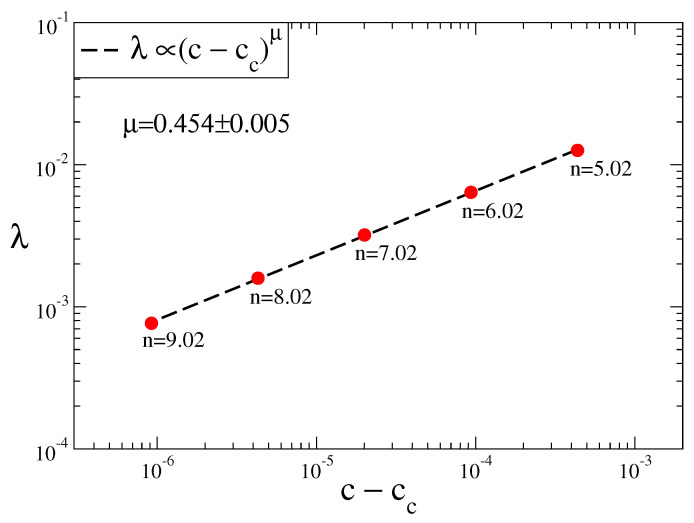
Scaling relation between the Lyapunov exponents of (n,c) tuples and their distance to the chaos threshold ($c-c_{c}$) for the Rössler system.

Huberman and Rudnick defined a universal expression for the parameter dependence of the Lyapunov exponent as the system moves from a chaotic state to the chaos threshold. With their definition, they showed that the Lyapunov exponent has the same form of scaling law as the critical phenomena describing the physical properties near the second-order phase transition in physics [33] if we consider the system transition from periodic behavior to chaotic. The Huberman–Rudnick scaling law is given by

(9) $$\lambda \propto {\left(c-c_{c}\right)}^{\frac{\ln2}{\ln\delta }}$$

where δ is the Feigenbaum constant and λ is the Lyapunov exponent [34]. For the Rössler system we calculate the Lyapunov exponents for (n,c) tuples and check the validity of the Huberman–Rudnick scaling law by plotting λ versus ($c-c_{c}$). In Figure 4 we observe that Lyapunov exponents represent a power-law scaling which is perfectly consistent with the universal Huberman–Rudnick scaling law.

### 3.2. Scaling Relation between the Correlation Length and Distance to the Chaos Threshold

**Figure 5:**
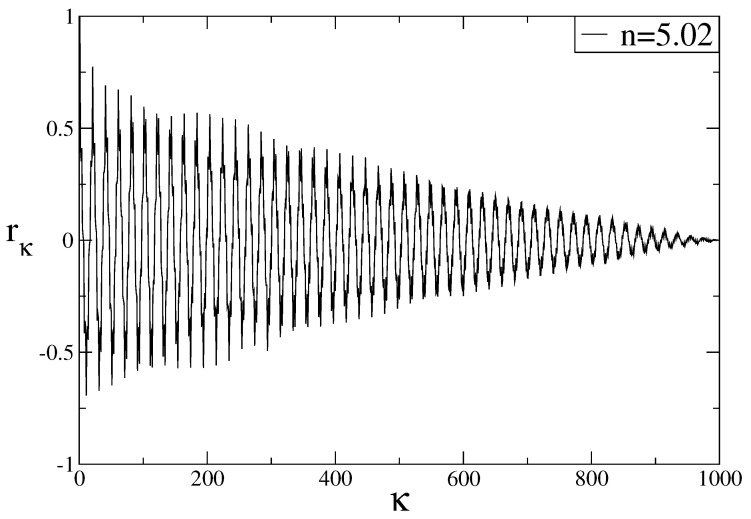
Auto-correlation function of data points obtained for the *y*-variable of the Rössler system for n=5.02.

Poincaré section points of the Rössler system in the vicinity of the chaos threshold show strong correlations due to strongly correlated data points in distinct bands. We study the correlation property of the data obtained from the Poincaré section of *x*, *y*, and *z* variables of the Rössler system. In order to analyze whether the data of a time series is correlated, we can use the auto-correlation function $r_{\kappa }$ which is defined as

(10) $$r_{\kappa }=\sum_{i=1}^{N-\kappa }\frac{\left(y_{i}-\langle y\rangle \right)\left(y_{i+\kappa }-\langle y\rangle \right)}{\sum_{i=1}^{N}{\left(y_{i}-\langle y\rangle \right)}^{2}},$$

where $\langle y\rangle =1/N\sum_{i=1}^{N}y_{i}$, *N* is the total number of data points of the time series, and κ is the time lag. The data is said to be correlated if $r_{\kappa }\neq 0$ and not correlated if $r_{\kappa }=0$. To visualize strong correlations of the Poincaré section points of the Rössler system, we plot the auto-correlation function (Equation (10)), of the data obtained from the Poincaré section that contains maximum *y* trajectories for n=5.02. As seen in Figure 5, the auto-correlation function of the data oscillates around zero with very large fluctuations.

As the chaos threshold is approached with increasing *n* values, correlations between trajectory points of $2^{n}$ chaotic bands get stronger. Due to this behavior, the correlation length ζ of a $2^{n}$ chaotic band and its distance to the chaos threshold can be related. ζ can be defined using the auto-correlation function $r_{\kappa }$ as

(11) $$\zeta =\sum_{k=1}^{K}\left(1-\frac{k}{N}\right)r_{\kappa }$$

where *K* is upper limit of the time lag κ (for κ<K, $r_{\kappa }\neq 0$ and κ>K, $r_{\kappa }=0$) corresponding to the correlated data [35]. In the calculation of the auto-correlation function (Equation (10)) and ζ (Equation (11)), we take the summation of the $N=2^{k}$ Poincaré points for each (n,c) tuple, (k=1,2,...,n). This summation of Poincaré points is defined as

(12) $$\eta :=\sum_{i=1}^{N}x_{i}=W_{k}.$$

Equation (12) is given for the Poincaré points obtained from the *x*-variable of the Rössler system, and for the other two variables the equation has the same form. As Poincaré points of the Rössler system behave in the same way as iteration steps of an iterative map, we fall back to our starting point at the chaotic band of our (n,c) tuple (not exactly to our starting point, but very close to it). Repeating this process $2^{k}$ times successively we cover the whole band and obtain $2^{k}$ strongly correlated variables $W_{k}$ possessing the length of $2^{k}$ [30,31].

**Figure 6:**
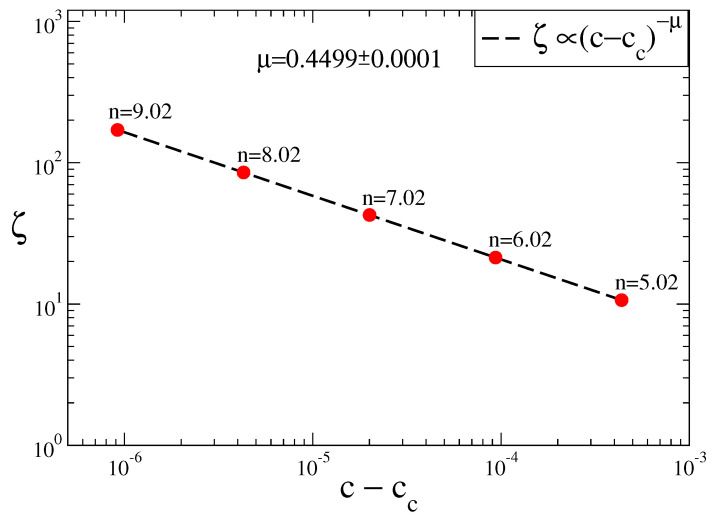
Plot of the correlation lenght of (n,c) tuples of the Rössler system ζ versus distances of the (n,c) tuples to the chaos threshold.

We plot the calculated correlation length of each (n,c) tuple versus their distances to the chaos threshold in order to obtain a scaling relation. In Figure 6 we see that as we approach the chaos threshold, ζ of $2^{n}$ chaotic bands increases with power-law behavior $\zeta \propto {\left(c-c_{c}\right)}^{-\mu }$ with μ=0.4499±0.0001 critical exponent value. This obtained scaling relation is valid for all of three variables of the Rössler system.

### 3.3. Analysis of the Fractal Dimension for the Rössler system

If we look from geometric point of view to the $2^{n}$ chaotic bands observed in the band-splitting procedure in the chaotic regime, we see that Poincaré points are approximately uniformly distributed on allowed regions of the phase space and there are forbidden regions among allowed ones. Due to the band-splitting procedure, the whole phase space has fractal properties [20,36].

In our study, for the fractal dimension calculation we use box-counting method that requires covering the phase space of the system with boxes of side length ϵ. We then fill these boxes with the iterations of the dynamical system. We use line segments of length ϵ since the Poincaré section points of the Rössler system construct a one-dimensional phase space and we fill these line segments with the Poincaré points. The box-counting fractal dimension is defined as

(13) $$D_{box}=\lim_{\epsilon \to 0}\frac{\log N(\epsilon )}{N_{box}}$$

where $N_{box}$ is the number of line segments needed to include all the points of geometric object for chosen ϵ length.

The band-splitting procedure in the chaotic regime can be seen as a Cantor set construction. From this perspective, in recent works [30,31] it has been shown for the one-dimensional logistic map that the fractal dimension of each $2^{n}$ chaotic band exhibits a power-law type scaling relation with its distance to the chaos threshold. At the most chaotic regime of the map, the whole phase space is fully occupied by iterations and can be regarded as a single band with $D_{box}=1$. This single band is involved in the first stage of the Cantor set construction and also the beginning of the band-splitting procedure [32].

As a consequence of the continuous system dynamics, the Rössler system has no analytical bounds for its phase space, unlike the logistic map. In the analysis of the fractal dimension for the Poincaré points of the flow, a problem emerges. Since there are no bounds for the phase space and parameter values, one may choose arbitrarily large control parameters, driving the system to the strongly chaotic regime. Before the first band is split, any case corresponds to a single band with $D_{box}=1$ which is fully occupied by Poincaré points. For this reason, choosing a band for the first stage of the Cantor set construction is arbitrary. Due to these difficulties arising from the nature of the flow, there is no scaling relation between fractal dimensions of $2^{n}$ chaotic bands and their distances to the chaos threshold for the Rössler system.

## 4. Conclusions

From the Poincaré section analysis of the Rössler system, we observe that a *q*-generalization of Pesin-like identity is valid at the chaos threshold and that there exist power-law type scaling relations in the vicinity of this critical point. For each variable of the Rössler system, the linear time dependence of the sensitivity function and the entropy increase rate are obtained for the same specific *q* value (q=0.36), showing that the generalization of the Pesin-like identity holds for this *q* value.

As Poincaré points obtained by the Poincaré section that collects maximum points of a signal of a variable of the Rössler system represent similar behavior to the one-dimensional unimodal dissipative logistic map, our results can be compared to the results that are given for the logistic map in [30,31]. In Table 2 we see that *q*-generalized Pesin-like identity is valid at the same *q* value for both systems. The same types of power-law behavior have also been observed for scaling relations as the chaos threshold of each system is approached.

As a result of the fact that the phase space of the flow is unlimited, the fractal dimension analysis cannot be handled in a similar manner to that of recent works [30,31] performed for the logistic map.

To the best of our knowledge, for the first time one of the paradigmatic model systems of flows (Rössler) is analyzed here in the realm of non-extensive statistical mechanics using the Poincaré technique. Obtained results have been compared to those of the paradigmatic one-dimensional dissipative map (a logistic map). Since these distinct systems share the same universality class, as expected the scaling relations have exactly the same exponents, which leads to the fact that λ∝1/ζ. The tendency of the Lyapunov exponent is exactly the same as that of the order parameter (magnetization) observed in the second-order phase transitions.

References

## Figures and Tables

**Table 1:** *n* Values that correspond to $2^{n}$ chaotic bands and *c* parameter values calculated from Equation (8) for these *n* values.

*n*	*c*
5.02	4.20466919339444...
6.02	4.20432584820760...
7.02	4.20425231418281...
8.02	4.20423656544653...
9.02	4.20423319254949...
*∞*	4.204232273304...

**Table 2:** Relations obtained for the Rössler system and the logistic map.

Relation	Logistic Map	Rössler System
$\lambda_{q_{sen}^{av}}=K_{q_{sen}^{av}}$	$q_{sen}^{av}=0.36$ [24]	$q_{sen}^{av}=0.36$
$\lambda \propto {\left(a-a_{c}\right)}^{\mu }$	μ=0.448±0.003 [30]	μ=0.454±0.005
$\zeta \propto {\left(a-a_{c}\right)}^{-\mu }$	μ=0.448±0.003 [30]	μ=0.4499±0.0001
